# Implications of increasing temperature stress for predatory biocontrol of vector mosquitoes

**DOI:** 10.1186/s13071-020-04479-3

**Published:** 2020-12-01

**Authors:** Mmabaledi Buxton, Casper Nyamukondiwa, Tatenda Dalu, Ross N. Cuthbert, Ryan J. Wasserman

**Affiliations:** 1 Department of Biological Sciences and Biotechnology, Botswana International University of Science and Technology, Palapye, Botswana; 2 Department of Ecology and Resource Management, University of Venda, Thohoyandou, 0950 South Africa; 3 GEOMAR, Helmholtz-Zentrum für Ozeanforschung Kiel, 24105 Kiel, Germany; 4 Department of Zoology and Entomology, Rhodes University, Makhanda, 6140 South Africa

**Keywords:** Biological control, Climate change, Critical thermal limits, Pest mosquitoes, Predator-prey interactions, Thermal tolerance

## Abstract

**Background:**

Predators play a critical role in regulating larval mosquito prey populations in aquatic habitats. Understanding predator-prey responses to climate change-induced environmental perturbations may foster optimal efficacy in vector reduction. However, organisms may differentially respond to heterogeneous thermal environments, potentially destabilizing predator-prey trophic systems.

**Methods:**

Here, we explored the critical thermal limits of activity (CTLs; critical thermal-maxima [CT_max_] and minima [CT_min_]) of key predator-prey species. We concurrently examined CTL asynchrony of two notonectid predators (*Anisops sardea* and *Enithares chinai*) and one copepod predator (*Lovenula falcifera*) as well as larvae of three vector mosquito species, *Aedes aegypti*, *Anopheles quadriannulatus* and *Culex pipiens*, across instar stages (early, 1st; intermediate, 2nd/3rd; late, 4th).

**Results:**

Overall, predators and prey differed significantly in CT_max_ and CT_min_. Predators generally had lower CTLs than mosquito prey, dependent on prey instar stage and species, with first instars having the lowest CT_max_ (lowest warm tolerance), but also the lowest CT_min_ (highest cold tolerance). For predators, *L. falcifera* exhibited the narrowest CTLs overall, with *E. chinai* having the widest and *A. sardea* intermediate CTLs, respectively. Among prey species, the global invader *Ae. aegypti* consistently exhibited the highest CT_max_, whilst differences among CT_min_ were inconsistent among prey species according to instar stage.

**Conclusion:**

These results point to significant predator-prey mismatches under environmental change, potentially adversely affecting natural mosquito biocontrol given projected shifts in temperature fluctuations in the study region. The overall narrower thermal breadth of native predators relative to larval mosquito prey may reduce natural biotic resistance to pests and harmful mosquito species, with implications for population success and potentially vector capacity under global change.

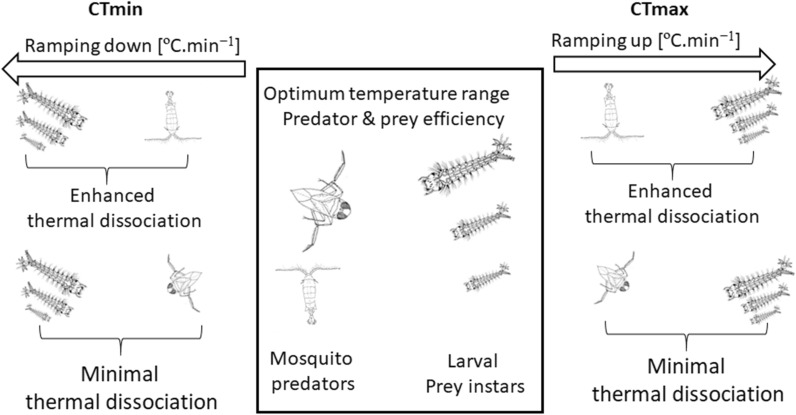

## Background

Population size in aquatic ecosystems is known to be largely dependent on ecological interactions such as competition and predation pressure [1, 2]. Predation plays a pivotal role in regulating problematic species (e.g. proliferating mosquitoes) through density-mediated effects, whereby population numbers are directly controlled through predatory removal or through indirect, trait-mediated effects such as compromised fecundity, growth rate and longevity of prey [3, 4]. Key mosquito genera (e.g. *Aedes*, *Anopheles*, *Culex*) are of public concern globally, transmitting pathogens that cause common debilitating diseases to humans (e.g. chikungunya, dengue, multiple kinds of encephalitis, elephantiasis, malaria, yellow fever, Zika), livestock (Rift Valley fever) and wildlife (avian malaria, West Nile) [5, 6]. Moreover, mosquitoes are semi-aquatic insects that colonize and develop (egg, larva, pupae) in aquatic habitats across natural, urban and peri-urban environments [7].

Naturally, mosquitoes coexist in aquatic microhabitats with a large faunal community [8], including aquatic predators that curb mosquito populations [9–14]. In these environments, both the predator and mosquito prey experience stressful thermal extremes, with variable effects on performance given differential thermal physiological responses [15, 16]. Mosquitoes breed in diverse, often cryptic, aquatic habitats such as rock crevices, phytotelmata (e.g. tree holes), animal hoof prints, artificial containers as well as larger-scale temporary and permanent water bodies [17, 18]. These environments are prone to extreme environmental fluctuations, which are expected to become the new norm in a warming world, typically becoming more intense, prolonged and frequent [19–21]. Mosquitoes have also adapted to colonize clean to highly compromised water quality sources, sunlit or shaded and of varying nutrient levels [22]. Predators of mosquitoes can persist in these environments as wholly aquatic organisms, access through aerial dispersal as semi-aquatic predators or be purposefully introduced as agents for desired ecosystem services [23]. The variable utilization of different water bodies by mosquito larvae, and their aquatic predators, has implications for performance of these organisms and ultimately predator-prey interaction outcomes. Understanding these dynamics could thus prove useful in determining the sustainability of natural and augmentative mosquito biological control in aquatic habitats, particularly within the context of changing environments [24].

Predator-prey interaction strengths can be mediated by abiotic environmental factors [25]. Particularly temperature is important in this regard, affecting organismal physiology, ecology, metabolism and overall fitness [26, 27]. Temperature in water bodies is essential as a regulatory mechanism that drives biochemical and physiological processes [28, 29], with implications for behaviour, performance and predator-prey interaction outcomes [30–32]. Indeed, empirical studies have shown environmental variability likely affects higher trophic levels, e.g. predators, more significantly than prey [33–35]. Furthermore, thermal performance is highly enigmatic and varies among species, ontogeny, age [36] and size [29]. Moreover, natural enemy efficacy also depends on the fate of bottom-up and top-down effects, which have been reported to favour pest and vector species [37]. As such, even slight alterations to temperature can compromise or heighten species fitness, community interactions and structure [38, 39]. This makes predicting the fate of natural enemy effects in the face of climate change highly complex. Additionally, at the autecological level, understanding how organismal critical thermal limits (CTLs) of varied species are affected by oscillating temperatures can be useful for broader ecological inferences, such as interaction dynamics between species [40, 41]. Definitively, CTLs represent temperatures at which an organism stops activity. Ecologically, activity here represents key fitness traits, e.g. mating, swimming and foraging ability [27, 42].

The effects of temperature are critical in determining the fate of trophic interactions under changing environments [43]. However, whilst studies have focused on terrestrial environments [34, 35, 44], to our knowledge, few have concerned aquatic habitats in the context of thermal tolerance, particularly for vector mosquitoes and their predators. Here, we aimed to assess thermal tolerance (lower and upper) of three regionally abundant aquatic mosquito predators (*Enithares chinai* and *Anisops sardea* [Insecta: Hemiptera], *Lovenula falcifera* [Copepoda: Calanoida]) and their vectorially important larval mosquito prey (*Aedes aegypti*, *Anopheles quadriannulatus*, *Culex pipiens* [Diptera: Culicidae]) in a semi-arid subtropical southern African landscape. All three mosquito species are commonly encountered in peri- and urban landscapes of the study region [45–47], with *Ae. aegypti* and *Cx. pipiens* well-known vectors of various pathogens that cause disease [48–50]. *Anopheles quadriannulatus* is not currently a known vector to human pathogens [51], however, is susceptible to *Plasmodium* infection [52, 53]. Physiological limits of this species may nevertheless serve as reasonable proxies of congeneric malaria vector species. While increases in extreme temperature events are predicted to be the future norm [19–21, 54–56], semi-arid southern Africa is projected to be particularly impacted by shifting climatic conditions [57]. We thus hypothesized that: (1) larval thermal tolerance would vary across mosquito species, with container-breeding specialists (principally *Ae. aegypti*) having the widest thermal window; (2) for predators, the wholly aquatic copepod would have the narrowest thermal window; (3) all predators would have narrower and therefore asynchronized thermal windows compared to the mosquito species.

## Materials and methods

### Animal collection and maintenance

Adult *Lovenula falcifera* and *Anisops sardea* were collected from a clay-lined temporary pond, Central District, Botswana (Fig. 2a; 022° 52′ 16.0 S; 027° 47′ 42.7 E), while *Enithares chinai* were collected from a concrete-lined water pool on the Botswana International University of Science and Technology (BIUST) campus (022° 35′ 46.8 S; 027° 07′ 30.5 E). The predators were housed in separate aerated 3-L plastic containers (covered with a net to prevent winged predator escape), comprising ~2 L of a 50:50 ratio of habitat water and matured tap water (kept for 48 h for dechlorination). These were placed in climate chambers (HPP 260, Memmert GmbH + Co.KG, Germany) set at 28 °C ± 2 and 65 % ± 10 relative humidity under a 12:12 light:dark photocycle. All predator species were fed *Cx. pipiens* larvae *ad libitum*. *Culex pipiens* larvae (accession number: MT741514) originated from egg rafts sampled from a concrete-lined water body situated in BIUST campus (022° 35′ 05.7 S; 027° 06′ 58.7 E). *Aedes aegypti* larvae (accession number: MK571449) were collected using a 1000 µm mesh net from a 20-L container holding ~10 L rain water in a homestead in Palapye village (022′ 32′ 97.6 S; 027′ 11′ 50.4 E) while *An*. *quadriannulatus* larvae (accession number: MT741513) were sourced from stagnant river water near Hogs Creek (022′ 34′ 79.3 S; 028′ 19′ 96.1 E). The larvae were reared, separately according to species, to different instar stages in 3-L plastic containers holding ~2 L matured tap water housed in climate chambers (as above) and fed with crushed rabbit food pellets *ad libitum* (Westerman’s Premium, Durban, South Africa). Both predators and their prey were kept at similar densities (10 individuals/L in a 3-L container holding ~2 L of a 50:50 ratio of habitat water and matured tap water) to avoid overcrowding effects on thermal fitness [58]. Both the predators and prey were collected between January and February 2020 and experienced similar thermal environments. Prior to all experiments, predators were kept for at least 7 days in laboratory rearing conditions while prey developmental stages were monitored until appropriate instars sizes were reached.

### Experimental design

We assessed CTLs in an experimental design with respect to (1) predators (3 species types: *L. falcifera, A. sardea*, *E. chinai*) and (2) mosquito larvae (3 species: *Cx. pipiens, Ae. aegypti*, *An*. *quadriannulatus*) across their instar stages (3 stages: mean length ± SE, early [1st instar; 1.5 ± 0.2 mm], intermediate [2nd/3rd instar; 3.0 ± 0.2 mm], late [4th instar; 5.1 ± 0.2 mm]). Critical thermal limits (CT_min_ and CT_max_) were measured randomly across all treatments, following modified protocols by Nyamukondiwa et al. [59]. A set of ten individual organisms at a time were each placed in a series of ten insulated double-jacketed chambers, connected to a programmable water bath (Lauda Eco Gold, Lauda DR.R. Wobser GMBH and Co. KG, Germany). The water bath contained a 1:1 water:propylene glycol ratio to sufficiently cater to sub-zero cooling temperatures. Each ‘organ pipe’ was filled with 50 mL matured tap water to house an individual animal, which was then given 10 min to stabilize at the 28 °C temperature, i.e. equivalent to climate chamber rearing conditions. A thermocouple (type T 36 SWG) connected to a digital thermometer (53/54IIB, Fluke Corp., USA) was inserted into a central ‘organ pipe’ (the control pipe) to monitor the water temperature experienced by the test animals. Temperature was ramped up (CT_max_) or down (CT_min_) (from benign 28 °C) at 0.25 °C min^−1^ following described protocols [59, 60]. The experiment was repeated twice (i.e. two runs per set of ten animals; *n* = 20) per treatment in keeping with Nyamukondiwa and Terblanche [61]. Here, we defined CTLs as the temperature at which an animal lost coordinated muscle function or responses resulting from a slight prodding using a thermally inert object congruous to Nyamukondiwa et al. [59]. For CT_max_, this loss of coordinated muscle function always coincides with lethal temperatures and mortality such that recovery is not possible. However, for CT_min_, recovery often occurs and thus the trait is not always lethal [27, 40].

### Microclimate data recordings

Microclimate temperature data were recorded from a sunlit (1) temporary clay-lined pond (123 m length × 95 m width × 1.5 m depth; 022° 52′ 16.0 S; 027° 47′ 42.7 E) and (2) temporary rock pool (2.4 m length × 1.7 m width × 13 cm depth; 22° 35′ 46.07″ S; 27° 07′ 16.46″ E), using programmable data logger probes and software (HOBOware Pro, version 3.7.16, Bourne, MA, USA) (0.5 °C accuracy; 1 h sampling frequency) during the period between August 2019 and February 2020. This was to determine the thermal fluctuations in these temporary wetland aquatic habitats that represent the dominant available natural breeding habitats for predators and mosquito prey in the region [62]. These temporary wetlands have been observed to host all larval species and the three predators tested. A data logger probe was placed on sediments at the bottom of the pond/pool during the dry period and monitored throughout the wet phases to reflect temperature variations associated with these environments, although species abundances were not monitored. The mean temperature for the wet phase, between both habitats, was used as the habitat temperature (*T*_hab_).

### Statistical analysis

Data analysis was performed using R, version 3.6.3. The residuals were first checked for normality and variance homogeneity using Shapiro-Wilks and Levene’s tests, respectively, and were found to violate normality and variance homogeneity assumptions. Therefore, a one-way Kruskal-Wallis nonparametric test was employed. The CTLs (CT_max_ and CT_min_) were considered separately as dependent variables, while the different prey species, instar stages and predator species were the independent factors. Statistically significant effects were examined pairwise post hoc using Dunn's test. We thus fit two models to our data, whereby the two CTLs (CT_max_ or CT_min_) of predators and prey (early, intermediate and late instars) were compared.

The thermal breadths (warming tolerance [WT] and cooling tolerance [CT]) for the predators and the larvae were calculated as described by Machekano et al. [63]:

$${\text{WT }} = {\text{ CT}}_{{{\text{max}}}} {-}T_{{{\text{hab}}}}$$

and,

$${\text{CT }} = T_{{{\text{hab}}}} {-}{\text{ CT}}_{{{\text{min}}}}$$

where CT_max_ and CT_min_ were the CTLs for the predators and larval prey whereas *T*_hab_ was the mean daily habitat (clay-lined pond and rock pool data combined mean) temperature, reflective of natural conditions likely experienced by both predators and the larval prey in these dominant temporary wetland environments. The standard errors (SE) between CTLs and the *T*_hab_ for each species were also calculated.

## Results

**Fig. 1:**
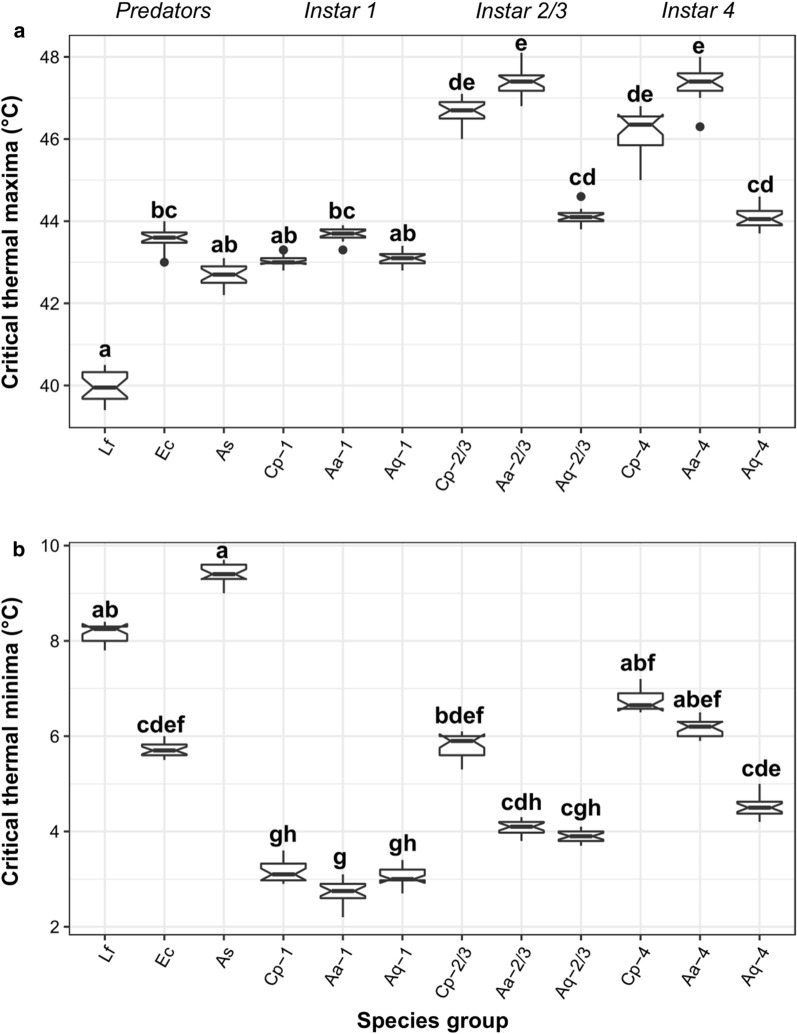
Critical thermal limits (**a** CT_max_ and **b** CT_min_) of three mosquito predators (*Lovenula falcifera* [Lf]*, Enithares chinai* [Ec] and *Anisops sardea* [As]) and three larval prey (*Aedes aegypti* [Aa]*, Anopheles quadriannulatus* [Aq] and *Culex pipiens* [Cp]) at the first, second/third and fourth instar stages. Group medians with different letters are statistically different from each other (*n* = 20 per experimental group). In the boxplots, the box gives the interquartile ranges and the whiskers show the largest and smallest values up to 1.5 × interquartile range. The horizontal line in each box shows the median

Critical thermal maxima (CT_max_) differed significantly among predators and prey across instar stages (*χ*^2^ = 229.43, *df* = 11, *p* < 0.001) (Fig. 1a). Among predators, *L. falcifera* exhibited the lowest CT_max_, which was the highest for the notonectids *E. chinai* and *A. sardea*, yet only significantly for *E. chinai*. Among prey, *Ae. aegypti* generally consistently exhibited the highest CT_max_, whilst *An. quadriannulatus* was the lowest and *Cx. pipiens* intermediate. Whilst the CT_max_ of first instar prey were all statistically similar, *Ae. aegypti* exhibited significantly higher CT_max_ than *An. quadriannulatus* at intermediate and late instar stages, but was statistically similar to *Cx. pipiens*. Within species, prey responses were in turn dependent on their instar stage, with first instar stages consistently exhibiting the significantly lowest CT_max_, whilst the later instar stages were not statistically different (Fig. 1a). As such, the extent of predator-prey CT_max_ mismatch was greatest when considering later instar stages.

Similarly, CT_min_ differed significantly across predator and prey types (*χ*^2^ = 233.63, *df* = 11, *p* < 0.001) (Fig. 1b). Among predators, the notonectid *E. chinai* exhibited the greatest cold tolerance (lowest CT_min_ temperature) compared to the other species, with CT_min_ significantly lower than *L. falcifera* or *A. sardea*. For prey, responses were inconsistent among species considering instar stage. For first instars, CTmin values were always statistically similar, whilst for second–third and fourth instars *Cx. pipiens* CT_min_ was the highest, and significantly higher than *An. quadriannulatus*. Within prey species, first instar stages consistently exhibited significantly higher cold tolerance (lower CT_min_), except between the first and second–third instars of *An. quadriannulatus* (Fig. 1b). Accordingly, in contrast to CT_max_, cold tolerance mismatches between predators and prey tended to be greatest for early instars.

**Fig. 2:**
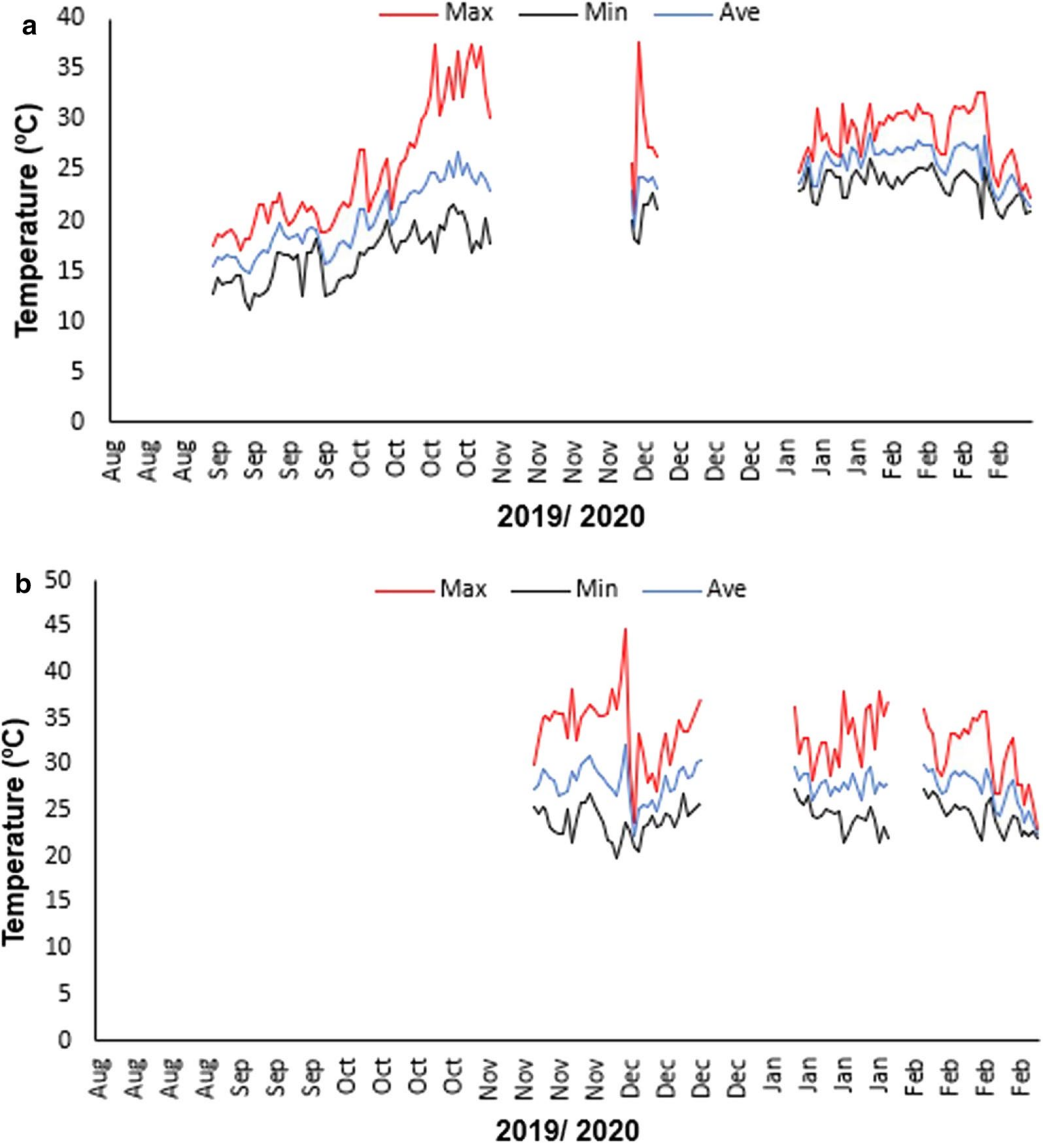
Microclimatic data showing mean daily maximum (Max), minimum (Min) and average (Ave) temperature (°C) of **a** a clay-lined pond and **b** a rock pool during their wet phase between August 2019 and February 2020

The mean daily *T*_hab_ obtained from the wet phases of the clay-lined and rock pool temporary wetland was 25.2 °C (minimum: 21.9 °C; maximum: 29.7 °C), and this was further used to determine the thermal breadths of mosquito predators and the larval prey (Table 1; Fig. 2). These results showed an overall trend of higher CTLs and wider thermal breadths for mosquito larval prey compared to their predators (Table 1). The notonectid *E. chinai* had the greatest thermal breadths compared to the other predator species at both temperature extremes. In turn, the WT of *L. falcifera* was narrower than that of *A. sardea*, but the same species (*L. falcifera*) exhibited a wider CT (Table 1)*.* Amongst larval mosquitoes, *Ae. aegypti* always had wider WT thermal breadths at matched instar stages. For CT, *Ae*. *aegypti* had the greatest breadths for the first instar stage alone; second/third and fourth instar stages were greater than in *Cx. pipiens*, but lower than in *An. quadriannulatus*. For all species, first instar stages had a narrower WT, but broader CT than later instars. Generally, aside from first instars, *An. quadriannulatus* had a narrower thermal breadth for WT and a wider CT breadth than *Cx. pipiens* (Table 1). *Enithares chinai* was an exception among predators, with wider WT and CT that was more similar to several larval mosquito stages (Table 1).

**Table 1:** Summary of thermal breadths (warming and cooling tolerance) of predators and larval prey calculated as temperatures between the critical thermal limits (CTLs) and the habitat temperature (*T*_hab_)

Species	Warming tolerance (°C)	SE	Cooling tolerance (°C)	SE
Predators				
* Anisops sardea*	17.53	± 0.49	15.77	± 0.48
* Lovenula falcifera*	14.83	± 0.59	17	± 0.43
* Enithares chinai*	18.41	± 0.54	19.43	± 0.39
Larval prey				
* Aedes aegypti* 1	18.5	± 0.39	22.41	± 0.48
* Aedes aegypti* 2/3	22.23	± 0.62	21.1	± 0.39
* Aedes aegypti* 4	22.21	± 0.62	19.01	± 0.42
* Anopheles quadriannulatus* 1	17.91	± 0.41	22.10	± 0.44
* Anopheles quadriannulatus* 2/3	18.94	± 0.42	21.26	± 0.34
* Anopheles quadriannulatus* 4	18.95	± 0.51	20.66	± 0.48
* Culex pipiens* 1	17.88	± 0.37	22.01	± 0.48
* Culex pipiens* 2/3	21.49	± 0.57	19.37	± 0.52
* Culex pipiens* 4	21.02	± 0.73	18.44	± 0.48

Means are shown alongside standard errors (SE)

## Discussion

Biological control of mosquito larvae using aquatic predators is a sustainable and environmentally friendly approach in reducing disease vector populations [64, 65]. Dissociations in thermal tolerances between predators and prey may, however, adversely affect predator-prey interactions leading to compromised foraging impacts. Our results showed varied thermal tolerance amongst mosquito larval prey, with early instars exhibiting compromised heat tolerance but having higher cold tolerance. Furthermore, the container-breeding specialist (*Ae. aegypti*) always had a wider WT (thermal breadth), indicating greater tolerance of heat stress. The wholly aquatic copepods had narrower thermal breadth, which was more compromised in terms of WT compared to other semi-aquatic notonectids. Overall, there was a mismatch between predators and the mosquito larval prey at both low- and high-temperature extremes. Predators showed lower thermal fitness and activity windows, suggesting that they may be impacted by temperature extremes earlier and more negatively than their mosquito prey. In keeping with Hunsicker et al. [66], these results potentially represent a loss of predator optimal ecosystem services provision and a mosquito larval prey proliferation cost in aquatic ecosystems with shifting environments. This has subsequent negative implications on increased vector population and associated disease risks amid rapidly shifting climate environments [67].

*Aedes aegypti* had a wider thermal window compared to the other mosquito species, having higher thermal limits to activity especially on the warming extreme. The species is highly invasive in tropical and subtropical regions of the world [46]. This could be linked to its behavioural profile of associating with thermally heterogeneous transient or temporary microhabitats, although CTLs may vary across mosquito species, space and methodological context [68, 69]. The species is found in human dwellings and thrives in diverse artificial containers as breeding habitats [70, 71]. Given that this species specializes in small water body utilization for oviposition (e.g. tyres, tins, gutters, flowerpots), *Ae*. *aegypti* have likely adapted (e.g. through transgenerational plasticity; [72]) and have the ability to withstand extreme temperatures in small environments associated with less thermal inertia. With the highest thermal tolerance and breadth compared to its predators and other mosquito prey larvae tested, *Ae. aegypti* has potential to thrive in conditions where other vector species would otherwise be compromised. Moreover, during this time, mismatch between *Ae. aegypti* and its predators could allow a rapid mosquito population growth owing to a lack of natural biotic suppression. This may further promote invasion success across the globe, with likely increased risks of associated pathogens and their diseases [73]. This has implications for emerging and reemerging diseases in vulnerable societies across rural-urban spheres with overall negative implications on public health and livelihoods. However, additional population regulatory effects of increased intraspecific competition should not be ignored.

Amongst the prey species assessed here, generally, *Cx. pipiens* was intermediate heat (CT_max_) and warming tolerant. *Culex pipiens* can breed in highly compromised water quality habitats (e.g. water treatment plants, sewage ponds, septic tanks) [74], and must oviposit directly onto water in contrast to *Ae. aegypti*, which produce dormant eggs. These environments provide a thermal variability cushion to the aquatic life stages compared to relatively smaller microhabitats that are likely not as buffered. Conversely, the results showed that *An. quadriannulatus* was generally the most cold tolerant, and generally least heat tolerant, except at first instars. Activity at low temperatures means *An. quadriannulatus* can still maintain key life history traits (e.g. swimming, foraging and development) during winter periods. Although the largely zoophilic *An*. *quadriannulatus* is not presently considered a malaria vector to humans [51], the species is susceptible to *Plasmodium* infection [52, 53]. This may have future implications for spatial emerging–re-emerging infections to humans, given potential shifts in behavioural [75, 76] and feeding preferences associated with such species [77, 78]. While *An*. *quadriannulatus* does not vector malaria, current thermal activity limit results are likely reasonable ecological proxies for more competent congeneric malaria vectors. To this end, we recommend further research considering thermal profiles of malaria-implicated vectors regionally, and diverse mosquito predators, alongside actual predator-prey performance experiments, in changing climatic conditions owing to the risk of emerging and reemerging diseases [79].

The results of this study generally reported that mosquitoes had wider thermal windows than their predators. Owing to this temperature regime mismatch, mosquito larvae are likely to proliferate against their predators, suggesting reduced efficacy of predator-prey interaction under shifting aquatic environments [80]. *Enithares chinai* heat tolerance (CT_max_) however synchronized with first instar stages of mosquitoes. However, Buxton et al. [14] showed that, at this size, there are some prey refuge effects, with the notonectid consuming second/third and fourth instar larvae more efficiently. This suggests that any observed thermal synchrony between *E. chinai* and first instar larvae may still offer limited biocontrol potential. Moreover, this study found that the wholly aquatic copepods are highly likely to be impacted in the warming temperature regimes as opposed to the semi-aquatic, air breathing predatory notonectids and the larval prey. Copepods’ life history traits are negatively affected by escalating temperatures as demonstrated by Lee et al. [81]. This has an overall implication for biocontrol in shifting environments, with the need to further identify diverse species, additively combine predators for sustainable mosquito regulation [82] and monitor (physiological-mediated traits) in time and space given the varied habitat temperature exposure. Although the species used here were a representation of a single location, the direction of the conclusions drawn is likely more broadly applicable for the studied taxa, all of which are widespread in southern Africa, and in certain instances other parts of Africa (e.g. *Lovenula* spp.) or even globally (e.g. *Ae. aegypti*). It would, however, be useful for future studies to investigate temperature effects across space and closely related taxa in search of unifying patterns. Similarly, future studies should also investigate predator-prey thermal interaction effects using more controlled predator and prey developmental stages (juveniles), since thermal fitness might vary across insect ontogeny. The relationship between CTLs and optimal temperatures also needs to be explored, within the context of predator-prey behaviour and interaction outcomes.

Based on the predator-prey dissociations exhibited here, whether predator thermal fitness can co-evolve symmetrically with their prey in changing aquatic environments remains a key question [83]. Field data have shown that, during the hydroperiod, performance of predators and mosquito larvae was within thermal breadths with no compromised activity on both extremes. Although pond temperature extremes could be seasonal and short-lived because of hydroperiods, more investigations on predator life history traits are warranted, especially the dormant egg physiology and hatching phenology consequent to the extreme hot and dry phases evidenced from clay-lined ponds and rock pools [84, 85]. Furthermore, the current study only measured basal thermal traits, and further exploration of other physiological-mediated traits driving the fate of predator-prey interactions within aquatic heterogeneous systems is needed. In particular, behavioural microclimate selection often drives invertebrate vulnerability to shifting climates [86]. Thus, the role of behaviour in modulating thermal fitness and how this may reshape predator-prey interactions also ought to be investigated.

Critical thermal limits have widely been used in assessing insect responses to climate change [27, 87, 88], including aquatic invertebrates [60, 89]. As such, these assays have gained attention in explaining the fate of trophic interactions, e.g. coevolved predator-prey and host-natural enemy association under high temperature stress [34, 35]. It is nevertheless critical to directly investigate and establish predator-prey interaction dynamics under varied temperatures [90, 91] and how optimal performance may relate to CTLs. This will allow for a more effective assessment of constraints of biological control associated with thermal stress prior to organismal loss of physiological function, e.g. through thermal performance curves (see discussions in [37]). While CTLs are only a measure of the fate of an organism at extreme temperatures [92, 93], they can still be useful in predictive models associated with population dynamics [27, 37, 94]. In addition, the outcomes have ecological implications not only for the long-term effects of global change [95], but also for the short- to medium term whereby organisms may be exposed to unexpected extreme acute temperatures, such as cold snaps and heat waves [21, 67, 96]. Although some organisms can survive these harsh conditions, some may succumb to them, with implications for community dynamics. In this context, although feeding rates of mosquito natural enemies can relate positively to temperature [97], as temperatures exceed thermal optimums a unimodal feeding relationship may arise [98, 99], which could alleviate prey from predation pressure and promote their proliferation.

Ectothermic organisms also often adaptively remodel their phenotypes to better survive stressful environments through plasticity [89, 100], a near ubiquitous mechanism in insects. Thus, it is likely that the limited thermal tolerance and breadths for predators recorded here may be compensated for through higher phenotypic adjustments [89, 101–103]. Nevertheless, the presence, magnitude and extent to which phenotypic plasticity may cushion organisms against climate change remains debatable [103, 104]. Thus, the exact extent to which plasticity may change the direction of interactions reported here warrants future investigation. Unraveling these physiological mechanisms will foster improved understanding of predator longevity and success, paramount to sustaining mosquito biological control under climate change. This is significant in maintaining the integrity and efficacy of biocontrol agent’s life history traits in aquatic habitats given the increased extreme temperature means and fluctuations with high intensities under global change [19, 20, 67, 104, 105].

## Conclusion

Our results demonstrate a mismatch of thermal activity limits (CTLs) and thermal breadths (WT and CT) between key predators and their mosquito prey. Larval mosquitoes had significantly higher activity limits and wider thermal windows relative to their predator antagonists. This thermal mismatch may mean asynchrony in predator-prey phenologies in shifting habitats, consequently altering the aquatic ecosystem trophic community structures and functioning. Predators are thus projected to reduce interactive foraging strength towards an increasingly thermally fit prey, giving vectors an advantage by proliferating in aquatic habitats. Implications for increasing temperature stress remain a challenge in predicting mosquito biocontrol using natural enemies, and more so under shifting aquatic habitats. In future research, the role of plastic thermal compensation in building resilience under climate change should be explored. Conservation of mosquito predators, coupled with the use of other complementary biological control strategies in an integrated approach may help reduce disease vector populations and associated public health concerns.

## Data Availability

The datasets used and/or analysed during the current study are available from the corresponding author on reasonable request.
